# Dataset for the estimation of a new body fat measurement method

**DOI:** 10.1016/j.dib.2020.106656

**Published:** 2020-12-23

**Authors:** Isaac Kuzmar, José Rafael Merlano Arroyo, Manuel Andrés Cantillo Villanueva, Laura Vanessa Sevilla Ortega, Gloria Sthefanny Goenaga Cortissoz, Xelenys Paola Granados Bandera, Eliana Zalabata

**Affiliations:** a Facultad de Ciencias de la Salud, Universidad Simón Bolívar, Colombia; b Fundación Universitaria del Área Andina, Valledupar, Colombia

**Keywords:** Body fat distribution, Obesity, Nutrition assessment, Ideal body weight

## Abstract

Due to the increasing prevalence of obesity and its negative consequences worldwide on public health, body composition analysis is a central pillar to assess the nutritional status. Scientists could use datasets to develop a new body fat measurement formula. Using bioelectrical impedance analysis, we analyzed the total body composition of 345 patients (234 men and 111 women) aged between 18 and 60 years residing in the metropolitan area of Barranquilla, Colombia. They have the potential for predictive formula analysis enhancing the cooperation among scientists. Due to the obesity pandemic, new datasets from other populations are needed to develop a body fat basic mathematical equation formula that could be used worldwide to determine the prevalence of overweight and obesity in a specific population group predisposed to develop metabolic syndrome or death, secondary to high cardiovascular risk.

## Specifications Table

**Table:** 

Subject	Health and medical sciences
Specific subject area	Biology
Type of data	Table Text Graph
How data were acquired	Physical Examination Bioelectric Impedance Analysis (BIA) technology
Data format	Raw Filtered
Parameters for data collection	Patient data was obtained from physical examination (height, weight, age, etc.) and body composition data was obtained using the BIA technology from Tanita; missing and atypical data were deleted, data was analyzed using SPPS Software.
Description of data collection	Data were obtained by physical examination of the participants and by a registered medical specialist using the with BIA technology (Tanita); all exploration was analyzed individually by a specialized software (Biological Suite Version 8). After getting data from all participants, the data were exported and tabulated for analysis in the SPSS software.
Data source location	Institution: BiomediKcal –Advanced Medical Nutrition &B Lifestyle Center City/Town/Region: Barranquilla Country: Colombia Latitude and longitude (and GPS coordinates, if possible) for collected samples/data: 10°58′6.74″ N −74°46′52.75″ W
Data accessibility	With the article Kuzmar, Isaac; Zalabata, orcid.org/0000–0002–3662–4371 (2020): DATASET FOR NEW BODY FAT MEASUREMENT. figshare. Dataset. https://doi.org/10.6084/m9.figshare.12982223.v2 https://doi.org/10.6084/m9.figshare.12982223.v2

## Value of the Data

• These data are important because they can serve as a model for future research allowing the development of new clinically useful basic mathematical formulas.
• Scientists can benefit from these data because it reports the datasets describing first body fat bioimpedance composition analysis that will be publicly available from a population of Barranquilla, Colombia.
• These data could be used for further insights and to develop experiments comparing the body composition of different populations and countries.

## 1 Data Description

Body mass index (BMI) is the method used to determine if the person has a weight within the normal range, i.e. whether a person is obese or thin. However, because it is an inaccurate measure, it is no longer used to evaluate the amount of body fat matter (body mass), represented by fat in the human body [1,2]. It is known that obesity is caused by excess fat mass in the body, so it greatly affects physical well-being [3]. This fat mass is considered a risk factor because it can manifest into multiple diseases, depending on the location of this fat mass [4]. Because other methods, such as DEXA and nuclear magnetic resonance, are very expensive, the electrical bioimpedance (the opposition of body tissues and fluids to the passage of electrical current) has become one of the most useful techniques to estimate body composition, both in research and clinically [5,6,7,8]. Hence, it is important to propose an accessible formula in the medical field that allows to quickly and safely estimate the body mass and to carry out the necessary medical or therapeutic interventions that can improve the quality of life.

The data presented in this article is from 345 patients (234 men and 111 women) aged between 18 and 60 years, residing in the metropolitan area of Barranquilla, Colombia. We collected data from the medical records obtained through medical–nutritional consultations of BiomediKcal–Advanced Medical Nutrition & Lifestyle Center of Barranquilla. Data were tabulated with different variables of interest for the research project, such as age, height, bioimpedance, electrical, fat mass, fat-free mass, among others and were provided in .sav and .csv format. Height in centimeters and bioimpedance values were obtained using the Tanita MC-780MA (Tanita Corp., Tokyo, Japan) medical equipment: BMI, fat mass, fat-free mass, basal metabolism, and bone mass.

We performed an initial statistical analysis between the subjects by gender (*N* = 345, *M* = 234 versus *F* = 111); average age of men was 39.2 years (SD 11.5) versus women 32.2 years (SD 10.0); mean height in men was 172.4 cm (SD 6.5) versus 160.2 cm (SD 4.7) in women; heavier mean weight in men versus women was found to be 60.3 kg, SD 7.4 in men and 59.2 kg, SD 6.5 in women. A higher BMI average was obtained in men versus women (24.5, SD 3.1 versus 23.8, SD 3.2). The mean basal metabolic rate was higher in men than women (1253.3 Kcal, SD 96.9 versus 1229.5 Kcal, SD 94.0). The average fat mass was 20.3 kg (SD 5.1), equivalent to a total fat mass percentage of 32.9% (SD 5.1). Further analysis in the development of the new body fat formula should demonstrate if there was a statistical significance between genders.

**Fig. 1:**
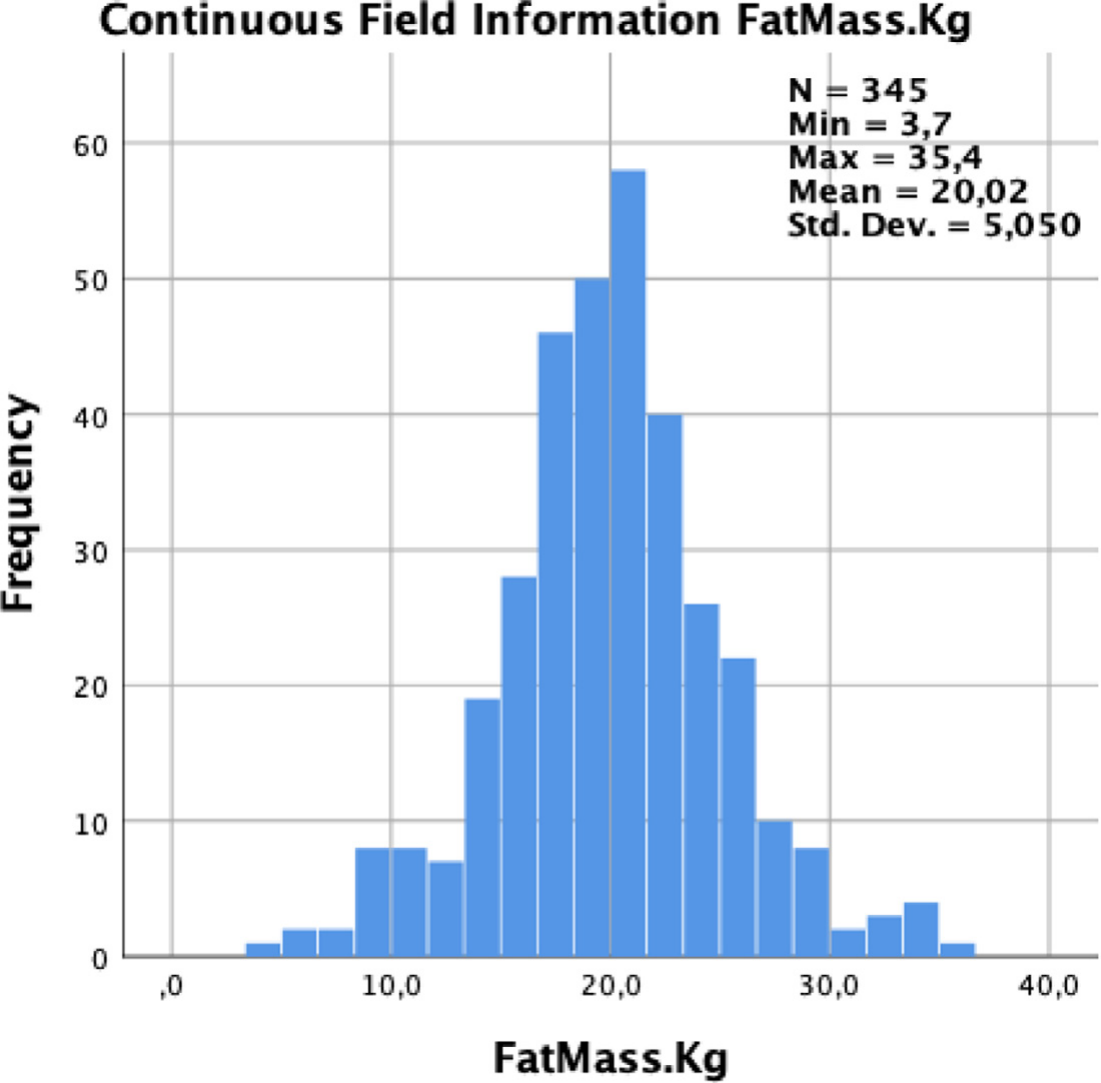
Graphic distribution of the Fat Mass in kilograms (mean= 20.0 kg, SD = 5.1).

Fig. 1 shows a graphical distribution of fat mass between genders with an average of 20.0 kg (SD 5.1) with a minimum of 3.7 kg and a maximum of 35.4 kg.

**Fig. 2:**
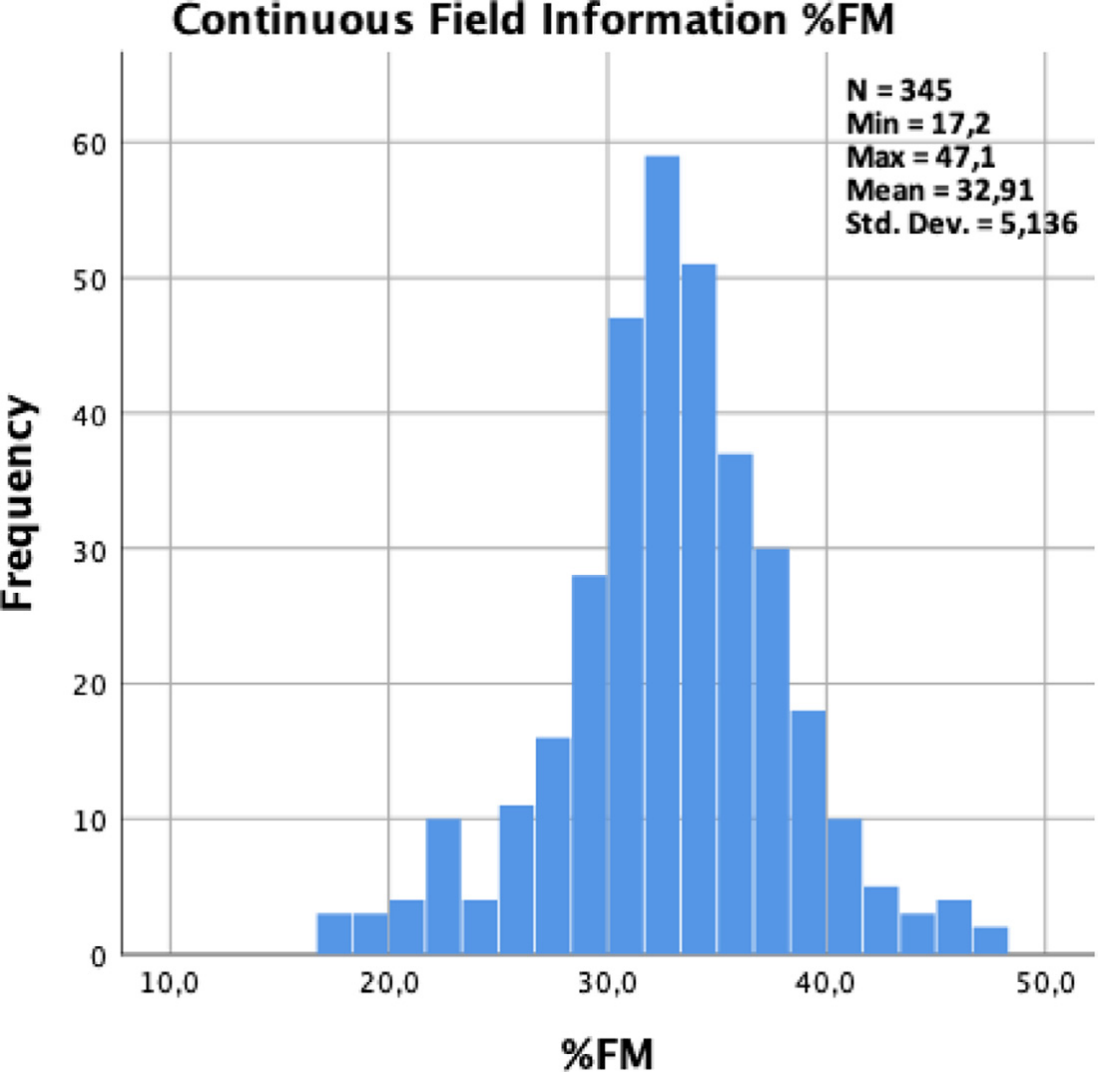
Graphic distribution of the Fat Mass in percentage (mean= 32.91%, SD = 5.14).

Fig. 2 shows a graphical distribution of the fat mass percentage with an average of 32.91% (SD 5.1) between genders, a minimum of 17.2% and a maximum of 47.1%.

## 2 Experimental Design, Materials and Methods

Experimental Design: Data were collected using a cross-sectional retrospective observational design.

Materials: Patients (*N* = 345, 234 male and 111 female) aged between 18 and 60 years residing in the metropolitan area of Barranquilla, Colombia were recruited in the study; data were collected from medical records obtained from medical–nutritional consultations in BiomediKcal–Advanced Medical Nutrition & Lifestyle Center of Barranquilla. Bioimpedance values were obtained using the Tanita MC-780MA medical equipment (bioimpedance device) with a specialized medical software. Data were analyzed using SPSS and Excel software. Stadiometer in centimeters.

Period: Data were collected from 2016 to 2019.

Inclusion criteria: Subjects older than 18 years, with desire for weight loss, desire to improve body image, and who signed informed consent were included in the study.

Exclusion criteria: Subjects who were pacemaker or metal orthopedic prosthesis carriers or women who were pregnant were excluded from the study.

Methods:

Height [9]: In a standing position and with no shoes, the patient was asked to remove any hair ornaments, jewelry, buns, or braids from the top of the head; then lean straight against the wall with the body weight evenly distributed and both feet flat on the floor with heels together and toes apart. Shoulder blades, buttocks, and heels touched the wall. With the head laid in the Frankfort plane (when the horizontal line from the ear canal to the lower border of the orbit of the eye was parallel to the floor and perpendicular to the vertical backboard), the stadiometer (SECA 206) was lowered to the head so that it rested firmly on top of the participant's head with sufficient pressure to compress the hair; the measurement and recording was to the nearest 0.1 cm.

Body composition analysis with impedance measurement: Weight and body compositions were determined using the Tanita MC-780 body composition analyzer (Tanita Corp., Tokyo, Japan) following the manufacturer's instructions.

All collected data was registered and tabulated in MS Excel and SPSS software for further analysis.

The Medical Subject Headings (MeSH) database was used when defining the keywords used in this article.

## Ethics Statement

The research was conducted according to the resolution number 8430 of 1993, which provides the scientific and administrative bases when conducting health research. Likewise, we followed the declaration of Helsinki issued by the world medical association, which gives the ethical principles that should guide the medical community and other people who are dedicated to conducting research with humans [10]. Table 1

**Table 1:** Group statistics according to gender.

Group Statistics				
	Gender(M:1;F:2)	N	Mean	Std. Deviation
Age	1	234	39,8	11,5
	2	111	32,2	10,0
Height.cm	1	234	172,4	6,5
	2	111	160,2	4,7
W.Kg	1	234	60,3	7,4
	2	111	59,2	6,5
BMI	1	234	24,5	3,1
	2	111	23,8	3,2
FatMass.Kg	1	234	20,2	5,0
	2	111	19,7	5,1
%FM	1	234	33,0	5,1
	2	111	32,8	5,3
FFM.Kg	1	234	40,1	3,4
	2	111	39,5	2,3
BMR.Kcal	1	234	1253,3	96,9
	2	111	1229,5	94,0

*N* = number of participants; *M* = male; *F* = female; Age in years; Height in centimeters; Weight in kilograms; BMI = Body Mass Index; Fat Mass in kilograms;%FM = Fat Mass Percentage; FFM.Kg = Free Fat Mass in kilograms; BMR.Kcal = Basal Metabolic Rate in kilocalories.

## CRediT author statement

All authors contributed equally.

## Declaration of Competing Interest

Dr. Kuzmar is co-founder of BiomediKcal–Advanced Medical Nutrition & Lifestyle Center of Barranquilla; he has a legal medical license, protects the anonymity of all his patients and any medical action he does is subject to surveillance by the Colombian medical ethics committee.
